# Further perspectives on measuring pulmonary oxygen uptake kinetics

**DOI:** 10.1113/EP091814

**Published:** 2024-02-26

**Authors:** Harry B. Rossiter, David C. Poole

**Affiliations:** ^1^ Institute of Respiratory Medicine and Exercise Physiology, Division of Respiratory and Critical Care Physiology and Medicine The Lunduqist Institute for Biomedical Innovation at Harbor‐UCLA Medical Center Torrance California USA; ^2^ Departments of Kinesiology and Anatomy & Physiology Kansas State University Manhattan Kansas USA

We thank Drs Francescato and Cettolo for stimulating further discussion on the ‘Independent breath’ (IND) algorithm for breath‐by‐breath gas exchange measurement. In Rossiter & Poole (2024) we mistakenly referred the reader to the work of Grønlund (1984) in relation to the basis for the ‘latter’ expiration‐only algorithm, rather than the intended ‘former’ IND algorithm. Thank you for correcting the record. For clarity, our reference to the *post ho*c application of breath‐by‐breath algorithms was to the original work, for example, of Grønlund (1984), and not to the IND approach (Francescato & Cettolo, 2023). Also, we suspect that Drs Francescato and Cettolo would agree that an expiration (typically) occurs between two consecutive inspirations, and therefore, that the $F_{O_{2}}$/$F_{N_{2}}$ (or $F_{CO_{2}}$/$F_{N_{2}}$) selected to indicate the start of a breath also occurs between consecutive inspirations.

Drs Cettolo and Francescato have previously quantified the effect of breath overlap using IND, where small fractions of gas exchange are double‐counted in consecutive breaths (i.e., they ‘become one’), and where algorithmic gaps between breaths result in an underlap or a gas exchange undercount. Errors in the volume of O_2_ exchanged were ∼4% during normal breathing at rest but rose to ∼22% during recovery from 10 s of hyperventilation (Cettolo & Francescato, 2018).

To our knowledge, most current commercial metabolic systems use an expiration‐only algorithm, providing accurate measurement of O_2_ uptake (${\dot{V}}_{O_{2}}$) at the mouth, without determining how much of that O_2_ is involved in alveolar–capillary exchange and how much is added to or released from lung gas stores. Therein lie the horns of two dilemmas. The first is to choose between an expiration‐only algorithm that is accurate (for ${\dot{V}}_{O_{2}}$ at the mouth) but where the degree of association with a variable of interest (alveolar ${\dot{V}}_{O_{2}}$) during cardiopulmonary exercise testing (CPET) is unknown; or the IND algorithm, which measures directly the variable of interest, but is known to introduce, potentially small, but non‐systematic error. The second dilemma is to choose between an expiration‐only algorithm that concurrently provides accurate measures of other clinically meaningful variables during CPET (Sietsema et al., 2020), such as respiratory exchange ratio, tidal volume (*V*_T_), breathing frequency, ventilation (${\dot{V}}_{E}$), deadspace fraction of a breath (*V*_D_/*V*_T_), ventilatory equivalents (${\dot{V}}_{E}$/${\dot{V}}_{O_{2}}$, ${\dot{V}}_{E}$/${\dot{V}}_{CO_{2}}$); or the IND algorithm where effects on these variables are unknown.

## AUTHOR CONTRIBUTIONS

Both authors have read and approved the final version of this manuscript and agree to be accountable for all aspects of the work in ensuring that questions related to the accuracy or integrity of any part of the work are appropriately investigated and resolved. All persons designated as authors qualify for authorship, and all those who qualify for authorship are listed.

## CONFLICT OF INTEREST

None declared.

## FUNDING INFORMATION

No funding was received for this work.
